# *Malassezia pachydermatis* Acquires Resistance to Polyenes in the Laboratory Model

**DOI:** 10.3390/pathogens14111162

**Published:** 2025-11-14

**Authors:** Urszula Czyżewska, Sandra Chmielewska, Marek Bartoszewicz, Adam Tylicki

**Affiliations:** 1 Department of Microbiology and Biotechnology, Faculty of Biology, University of Bialystok, K. Ciolkowskiego 1J, 15-245 Bialystok, Poland; mbartosz@uwb.edu.pl (M.B.); atyl@uwb.edu.pl (A.T.); 2 Doctoral School, University of Bialystok, K. Ciolkowskiego 1K, 15-245 Bialystok, Poland; s.chmielewska@uwb.edu.pl; 3 Metabolomics and Proteomics Laboratory, Clinical Research Centre, Medical University of Bialystok, Sklodowskiej 24a, 15-276 Bialystok, Poland

**Keywords:** antimycotics, natamycin, nystatin, yeasts

## Abstract

This study presents a model investigation into the development of tolerance to polyene antifungal drugs (nystatin and natamycin) in strains of *Malassezia pachydermatis*. This species, commonly associated with external ear canal infections in dogs, has emerged as increasingly significant in the broader context of growing fungal resistance to treatment. In the experiment, 10 strains of *M. pachydermatis* were passaged over a period of 105 weeks on media containing sublethal concentrations of nystatin and natamycin. Minimal inhibitory (MIC) and minimal fungicidal concentration (MFC) values were regularly assessed to monitor tolerance development. The results revealed a varied response among the strains: Some were eliminated during the process, while others showed a gradual increase in MIC values, up to fivefold in the case of nystatin. In several strains, acquired resistance remained stable even after passaging in drug-free conditions, whereas others reverted to their original susceptibility. The model demonstrated that resistance does not emerge immediately; significant changes appeared only after 30–45 passages. The authors propose this model as a valuable tool for tracking sequential changes that lead to resistance development. Such an approach may support targeted therapy development and help identify strains predisposed to drug adaptation. These findings hold promise for assessing therapeutic risk in immunosuppressed patients and for building resistance datasets that can support artificial intelligence algorithms in predicting fungal resistance mechanisms.

## 1. Introduction

The epidemiology of infections caused by *Malassezia* spp. predominantly pertains to dermatological infections of companion animals such as dogs and cats. Species of the genus *Malassezia* are commonly found on human skin, with their presence reported in more than half of the population, particularly in warm and humid climates, where they are often implicated in superficial infections such as pityriasis versicolor [1,2]. There have also been reports of systemic infections caused by *Malassezia* spp., primarily affecting high-risk patient groups such as premature neonates and elderly individuals receiving parenteral nutrition [3,4,5,6,7]. Infections due to *Malassezia* spp. often manifest as chronic and requiring prolonged antifungal treatment, thereby contributing to the emergence of antifungal resistance—a pressing challenge in contemporary biology, medicine, and veterinary science [8].

*Malassezia pachydermatis* is a common commensal of the skin and mucous membranes of animals, mainly dogs. It is the most frequently identified etiological agent in external otitis (otitis externa; 30–80% of cases) and dermatitis (30% of cases), which are commonly characterized by seborrhea, pruritus, and erythema [9,10]. These conditions are frequently associated with immunological disorders, hypersensitivity reactions, keratinization defects, or anatomical predispositions such as skin folds or stenosis of the auditory canal [1,2,11,12]. Screening studies indicate that this species also inhabits the skin of 20% to as much as 80% of healthy animals [13,14]. Ziółkowska and Nowakiewicz found the presence of *Malassezia* yeasts (mainly *M. pachydermatis*) in as many as 81% of cases of otitis in dogs [15]. The presence of *M. pachydermatis* in humans is rather transient, and it is rarely isolated from healthy human skin. Nevertheless, *M. pachydermatis* may be an example of a zoophilic opportunist that is increasingly associated with human disease. In humans, in addition to skin lesions, it can cause systemic infections in cases where the host’s immunity is compromised and in neonates hospitalized in intensive care units receiving parenteral nutrition [16,17,18,19,20]. The first case of systemic mycosis caused by *M. pachydermatis* was described by Fine et al. in 1983 in a patient with type 1 diabetes receiving outpatient peritoneal dialysis [21]. The literature indicates that healthy people (e.g., medical personnel with dogs) may serve as vectors for transmitting *M. pachydermatis* to hospital patients. Individuals on immunosuppressive therapy and newborns are particularly vulnerable to such infections [16,17].

Currently, there are five major classes of antifungal agents, of which azoles, echinocandins, and polyenes are the most commonly used [22]. Finding new antifungals remains exceptionally challenging due to the eukaryotic nature of fungal cells and their metabolic pathways, which closely resemble those of human cells. This biological similarity significantly raises the risk of potential new drug toxicity, thereby limiting the clinical applicability. Among the above-mentioned, polyenes represent one of the oldest and most extensively utilized classes since the mid-20th century. These macrolide compounds are naturally produced by *Streptomyces* spp. and are employed in the treatment of both invasive systemic mycoses (e.g., amphotericin B) and superficial infections (e.g., nystatin, natamycin) [23,24,25]. Polyenes play a critical role in the treatment of fungal infections, given the increasing resistance to other antifungal classes such as azoles and echinocandins. Fungal strains resistant to ergosterol biosynthesis inhibitors (e.g., azoles) often remain susceptible to polyenes, as the latter act through ergosterol binding rather than interference with its biosynthetic pathways [26,27].

**Figure 1:**
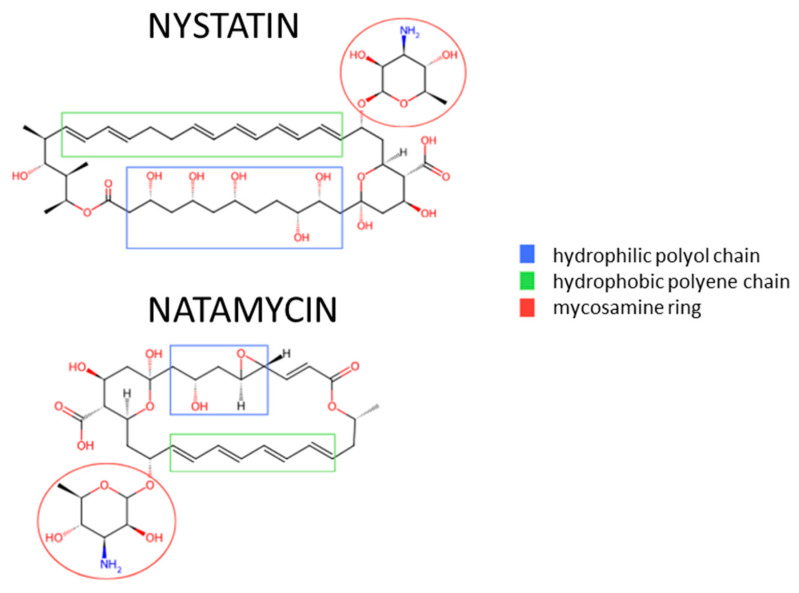
Comparison of the chemical structures of nystatin and natamycin [27,28].

Nystatin and natamycin (Figure 1) mainly differ in the length of the hydrophilic polyol chain and hydrophobic polyene chain composed of a conjugated double-bond system. These structural differences affect both their mechanisms of action and their clinical application. The antifungal mechanism of nystatin involves direct binding to ergosterol in the fungal cell membrane. This interaction leads to the formation of hydrophilic pores, resulting in the disruption of the membrane and cell death. In contrast, natamycin, due to its specific chemical structure, diminishes its ability to form pores in fungal membranes by limiting its affinity for ergosterol. Consequently, natamycin exhibits a different mode of action—it induces oxidative damage in the membrane, thereby impairing the integrity and functionality of the fungal cell membrane [28]. Some studies have also shown that polyenes can have a direct inhibitory effect on the function of membrane transporters for certain amino acids and glucose. Initially, this was attributed to nonspecific processes, such as the dissipation of transmembrane ion and substrate gradients due to membrane permeabilization. In fact, these transporters are inhibited by the impermeable polyene natamycin, but the exact mechanism is not yet fully understood [28,29].

Antifungal resistance has been documented across all known classes of antifungals. Frequent and often inappropriate use of antifungal drugs contributes significantly to the selection of resistant strains [30]. Subtherapeutic dosing, which may promote fungal adaptation, increases the risk of resistance development [31,32]. Environmental contamination with pharmaceutical waste, including the presence of antifungal agents in water and soil, creates selective pressure that fosters the emergence and dissemination of antifungal resistance [33]. Globalization, along with the increased mobility of people and goods, has facilitated the worldwide spread of resistant fungal strains [34].

Another critical factor is the extensive use of fungicides, which are structurally similar to antifungal drugs, in agriculture and the food industry. Natamycin, for instance, is employed as a food preservative [35]. Its use is restricted to the production of aged cheeses and cured dry sausages. The maximum permitted concentration of natamycin is 1 mg/dm^2^ of surface area, and it must not be detectable beyond a depth of 5 mm within the product [36].

The number of patients affected by fungal infections continues to grow each year. In immunocompromised individuals, these infections frequently result in fatal outcomes. At the same time, the increasing resistance of yeasts to the limited arsenal of available antifungal drugs poses a critical problem. Therefore, the investigation of resistance mechanisms is of paramount importance, particularly since our understanding of this phenomenon in fungi remains incomplete. Advancing research in this area may lead to the development of more effective therapeutic strategies and, in the long term, enable the identification of strains with a high propensity to develop resistance to specific classes of antifungal compounds. *Malassezia* spp. provide a compelling example: although they are common components of the human microbiota, under certain conditions, such as immunosuppression, they can become pathogenic and increasing resistance in these yeasts may pose a significant future challenge, particularly in immunocompromised patients [7,12].

Taking into account the above information, our study aimed to establish an experimental model for the acquisition of antifungal resistance to selected polyenes (nystatin, natamycin) through prolonged exposure to sublethal concentrations of these agents in initially susceptible *M. pachydermatis* strains. This methodical approach to the problem of drug resistance differs from the majority of studies reported in the literature, which typically infer resistance mechanisms by comparing resistant and susceptible isolates [37,38,39]. Instead, our approach allows for direct observation of the changes occurring during the stepwise development of resistance in initially susceptible strains. This future provides the opportunity to reconstruct the chronological sequence of events underlying the acquisition of resistance and a better understanding of the process of increasing tolerance to antibiotics.

## 2. Materials and Methods

*M. pachydermatis* strains used in this study came from a collection of the Laboratory of Cytobiochemistry, Department of Microbiology and Biotechnology, Faculty of Biology, University of Bialystok. The isolates (n = 63) were obtained from the external auditory canal of dogs, both those clinically diagnosed with otitis externa (PU) and asymptomatic animals (Z) [40]. All *M. pachydermatis* isolates were genotypically identified using the ITS-RFLP method (Intergenic Transcribed Spacer—Restriction Fragment Length Polymorphism) [41]. For *M. pachydermatis*, this method involves PCR amplification of the ITS region of ribosomal DNA, followed by digestion of the PCR products (800 bp) with restriction enzymes (*EcoRI* endonuclease). The resulting DNA fragments are separated electrophoretically, yielding a characteristic banding pattern (350 bp and 450 bp) that reflects sequence polymorphisms within the ITS region and allows for differentiation of fungal species.

To assess the initial susceptibility of all 63 *M. pachydermatis* strains to nystatin and natamycin—as well as during subsequent stages of model development—minimal inhibitory concentration (MIC) and minimal fungicidal concentration (MFC) assays were performed using a broth dilution method, adapted from the CLSI guidelines (Clinical and Laboratory Standards Institute, M27-A4-2017) [42].

MIC assay. For MIC determination, each strain was pre-cultured for 72 h on YPDA medium (1% yeast extract, 2% peptone, 2% dextrose, 2% agar) at 32 °C. Subsequently, suspensions of each strain were prepared in 0.9% NaCl to a turbidity of 0.5 McFarland standard (corresponding to 1.5 × 10^8^ cells/mL). In the next step, 10 µL of inoculum was added to 1.5 mL Eppendorf tubes containing 1 mL of liquid YPD medium supplemented with either natamycin (Merck Life Science, Darmstadt, Germany) or nystatin (Merck Life Science, Darmstadt, Germany) across a concentration gradient ranging from 0 to 90 µg/mL in 2 µg/mL increments. MIC values were determined after 7 days of incubation at 32 °C as the lowest concentration of antifungal agent at which no visible yeast growth was observed. All MIC values were established based on three independent replicates.

MFC assay. The determination of minimal fungicidal concentration (MFC) involved spotting 3 µL aliquots of the experimental cultures prepared for MIC assessment onto solid YPD medium without antifungal agents. Each sample was plated in triplicate. The inoculated plates were incubated at 32 °C for 7 days. The MFC was defined as the lowest antifungal concentration at which no visible growth of *M. pachydermatis* was observed. MFC values were determined based on three independent replicates.

Strain selection criteria. Based on the MIC and MFC values of nystatin and natamycin for all *M. pachydermatis* isolates, strains were selected according to their suitability for establishing the antifungal resistance acquisition model. Ten isolates displaying the highest susceptibility to both polyenes (i.e., the lowest MIC values for both nystatin and natamycin; Table 1 and Table 2) were chosen for further experiments. Each selected strain was divided into two experimental variants: one passaged on YPD medium supplemented with nystatin (NYS variant), and the other on YPD medium with natamycin (NAT variant).

Monitoring resistance acquisition. All selected strains were incubated at 32 °C and subcultured every 7 days on solid YPD medium supplemented with either nystatin or natamycin for a total duration of 105 weeks. The concentrations of antifungal agents used in the media were set to remain below half of the average MIC determined for the selected strains (4 μg/mL for nystatin and 8 μg/mL for natamycin). MIC and MFC values for both polyenes were reassessed every 15 passages according to the previously described procedures. At these time points, samples from each culture were collected and stored at −80 °C in liquid YPD medium containing 10% glycerol.

## 3. Results

Among the strains selected, several were naturally eliminated during the experiment. In the NYS variant, four strains (*Z15*, *Z63*, *5PU*, and *46PU*) were unable to grow in the presence of the applied antifungal concentrations. In the NAT variant, one strain (*Z8*) failed to grow under experimental conditions. All of these strains showed growth on media supplemented with polyenes only up to 45 passages. The remaining strains displayed highly variable responses to the presence of polyenes in the culture medium:

- The highest increase in MIC values for nystatin was observed in the case of *Z27* and *28PU* strains (a 5-fold increase compared to initial MIC values), and for natamycin in strains *46PU* and *Z15* (a 2-fold increase).
- A decrease in MIC compared to baseline was observed in the NAT variant for strain *5PU*.
- Strain *Z28* showed no change in natamycin sensitivity throughout the experiment.

In most strains selected for the experiment, MFC values corresponded closely to MIC values in both experimental variants (Table 1 and Table 2).

**Figure 2:**
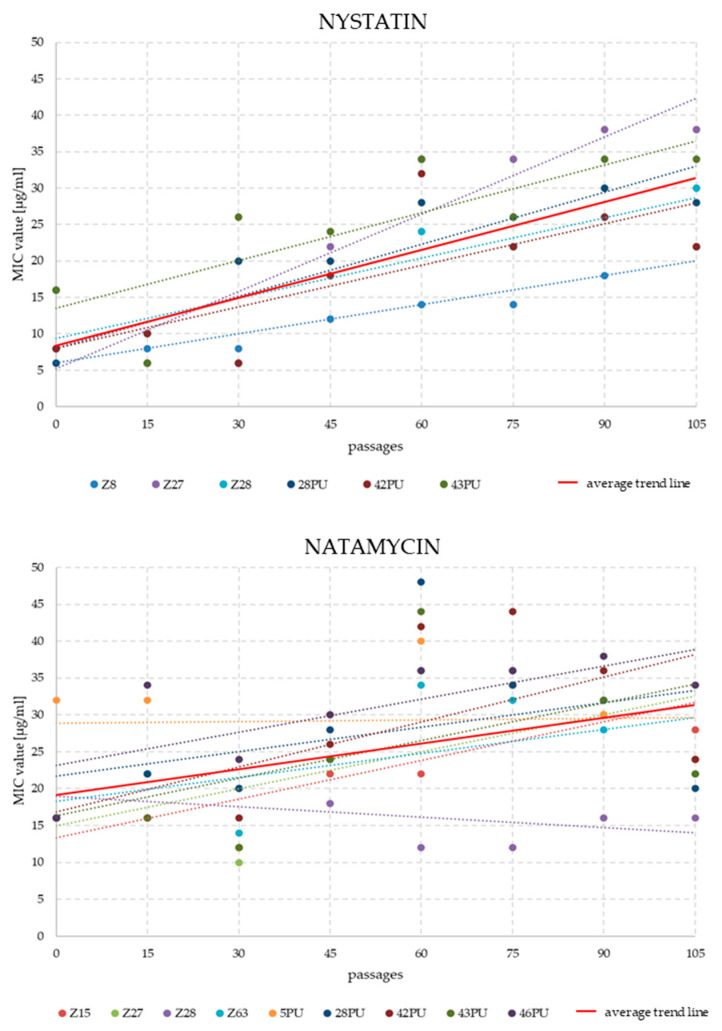
Trend lines of MIC value changes for nystatin and natamycin in individual *M. pachydermatis* strains (only those that survived until the end of the experiment) over 105 weekly passages on antifungal-supplemented media (4 μg/mL for nystatin and 8 μg/mL for natamycin).

**Figure 3:**
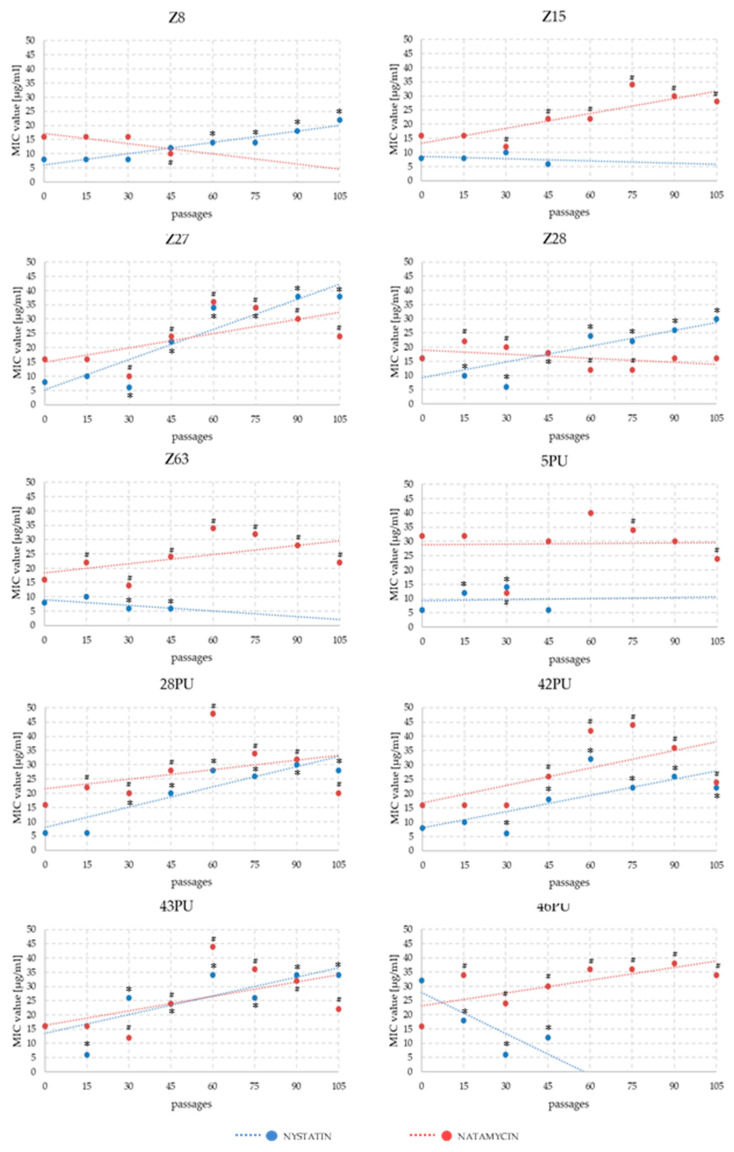
Trend lines of MIC value changes for nystatin and natamycin in *Malassezia pachydermatis* strains across successive passages during experimental modification of antibiotic sensitivity. Statistically significant differences compared to the initial state of * nystatin and ^#^ natamycin, *p* < 0.05, ANOVA, post hoc Tukey’s HSD test.

Analysis of MIC changes for nystatin and natamycin across successive passages revealed that resistance development was not immediate. Significant differences typically emerged between passages 30 and 45. In some strains, an initial decline in MIC values was followed by a gradual increase over several subsequent passages. The highest MIC values for natamycin were recorded between passages 60 and 75. By the end of the experiment, MIC values had fallen slightly. In the NYS variant, for strains maintaining growth until the end of the experiment, a systematic increase in MIC values was generally observed (Figure 2 and Figure 3).

To assess the stability of acquired polyene tolerance, after 105 passages of the main experiment, all strains were maintained for 10 weekly passages on YPDA medium without antifungal agents. Subsequently, MIC and MFC values for nystatin and natamycin were re-evaluated (Table 3 and Table 4). The results indicated that not all strains retained the same level of resistance observed at passage 105 of the main experiment. Resistance in the NYS variant appeared more stable. In contrast, most strains with the NAT variant reverted to baseline MIC values or to values approximating the initial susceptibility.

## 4. Discussion

Antifungal resistance modeling highlights the dynamic and rapid acquisition of resistance, particularly to azoles, which remain one of the most widely used antifungal drug classes. While polyene resistance has historically been considered rare, it is now emerging as a growing concern, especially in the context of multidrug-resistant pathogens such as *Candida auris*, which spreads efficiently in healthcare settings and presents a significant threat to public health. It is important to emphasize that current resistance models are constrained by a focus on a limited number of well-characterized species, such as *Candida albicans* or *Aspergillus fumigatus*. This narrow perspective hinders the generalization of findings and limits our understanding of resistance mechanisms in other clinically relevant fungi. Expanding in vitro studies to encompass a broader range of fungal species is therefore essential. An example is the *Malassezia* genus, which, although a common component of the human microbiota, can become pathogenic under certain conditions (e.g., immunosuppression). The increasing resistance of these yeasts to available antifungal drugs may become a significant problem in the future, particularly in immunocompromised patients. Therefore, our major advantage is the choice of an opportunistic yeast species, *M. pachydermatis*, a common pathogen of warm-blooded animals.

One of the key approaches to better understanding fungal drug resistance is the modeling of resistance development under controlled laboratory conditions. Such experiments yield valuable data at both the genetic and metabolic levels, enabling the identification of critical features associated with adaptation and resistance. These studies allow for the elucidation of mechanisms that enable fungi to survive in the presence of pharmacological agents and the identification of metabolic and genetic patterns characteristic of strains developing tolerance. A common strategy of drug resistance research in most cases involves comparative analyses of resistant versus susceptible strains but does not take into account the variability of the compared strains. This strategy facilitates the rapid identification of resistance-associated traits but makes it impossible to study the changes that occur in individual strains during the acquisition of resistance [37,38,39]. In our study, we adopted an alternative strategy based on modeling resistance acquisition by cultivating initially susceptible strains in the presence of antifungal agents to derive resistance development. This approach brings a model for future detailed tracking of the complex process by which fungal pathogens acquire resistance over successive stages, which closely mirrors the evolutionary dynamics of strains observed in natural or clinical environments. Most existing resistance acquisition models have been developed for *Candida* species, particularly *C. albicans* [43,44,45,46,47,48,49,50]. Only a limited number of studies have focused on other species such as *Saccharomyces cerevisiae* or *Aspergillus flavus* [51,52]. Despite their significance, such laboratory experiments are time-consuming, which limits the availability of corresponding data in the literature. In practice, these studies frequently emphasize genetic alterations, often overlooking equally important phenotypic changes, such as metabolic adaptations or biofilm structure modifications. Nonetheless, data generated through such experimental models provide an essential contribution to the growing antifungal resistance databases, which in turn support the development of machine learning (ML) and artificial intelligence (AI) algorithms for more accurate modeling and prediction of resistance mechanisms. The integration of large-scale datasets encompassing both genetic and metabolic dimensions is crucial for designing effective strategies to mitigate antifungal resistance. Developments in ML and AI technologies enable the analysis of large biological datasets, such as gene expression profiles in response to drugs, mutational patterns, and metabolomics [53]. ML algorithms can identify adaptive patterns that indicate the risk of resistance. Examples include analyzing the expression patterns of genes encoding active drug efflux pumps or enzymes involved in the response to oxidative stress, often associated with the action of antifungal drugs. Practical applications of these technologies include incorporating analysis of resistance gene mutations into routine testing of clinical strains, enabling early therapy adjustments and the design of new drugs by identifying molecular targets less susceptible to mutations [53,54]. Additionally, the development of rapid phenotypic and genotypic tests that enable the detection of resistance-associated mutations or metabolic changes could be a key step in personalizing treatment. Particular attention is currently being paid to the metabolic characteristics of fungal strains that may predispose them to resistance. Metabolomic analyses, which allow for the study of the complete profile of cellular metabolites, enable the identification of metabolic pathways associated with adaptation to environmental pressures, including drug exposure [55]. Understanding metabolic adaptive traits and the development of analytical technologies provides new opportunities for predicting resistance and designing more effective therapeutic strategies. The literature indicates the need for further investigation of these processes, particularly in the context of highly adaptive fungal strains such as *C. auris* and strains of the genus *Aspergillus* [56,57]. So, for this reason, we selected the opportunistic yeast *M. pachydermatis* as the subject of our investigation. Our study contributes novel insights into this species, which has remained relatively understudied in the context of resistance development.

The first compounds used in attempts to induce antifungal resistance in laboratory conditions in *Candida* species were nystatin and amphotericin B; however, these early efforts proved unsuccessful because authors could not obtain a stable resistance model [56,57,58,59,60]. Our findings confirm that not all strains are capable of developing resistance to a given antifungal agent, or even surviving on media containing its long-term sublethal concentration. In our long-term experiment, one out of ten strains failed to withstand the selective pressure of sublethal concentrations of natamycin, while four strains were eliminated due to their inability to grow in the continuous presence of nystatin. These results indicate the great importance of the unique characteristics of individual strains in the potential for the development of drug resistance. If so, the data also indicate the possibility of identifying characteristics of strains with a high potential for acquiring resistance. In prospect, revealing such traits may be crucial in evaluating a strain and taking appropriate therapeutic decisions.

In 1965, Hebeka and Solotorovsky reported the successful induction of amphotericin B resistance in *Candida albicans* (a 60-fold increase in MIC compared to baseline), as well as a moderate increase in tolerance to nystatin (a two- to threefold MIC increase, depending on the induction method), and observed cross-resistance between these two polyenes [43]. In our experiment, we obtained two strains in which nystatin tolerance increased approximately fivefold. However, regarding the development of cross-resistance, our results demonstrated that *M. pachydermatis* strains did not respond uniformly to both drugs (Figure 2). Four strains exhibited increased MIC values for both antifungal agents, while in five strains, MIC values increased for one compound while decreasing for the other.

In 2021, Carolus et al. reported the emergence of *Candida auris* populations with reduced susceptibility to amphotericin B during an experimental model of multidrug resistance induction [50]. In 1971, Athar and Winner screened 2000 clinical isolates of *Candida* spp. and found no evidence of constitutive resistance to nystatin or amphotericin B [44]. However, they succeeded in inducing significant in vitro tolerance to both drugs, surpassing the levels observed by Hebeka’s group. Interestingly, both studies reported that resistance acquisition was accompanied by a reduction in virulence [43,44]. In more recent investigations on polyene resistance induction using natamycin in several *Candida* species (*C. albicans*, *C. krusei*, and *C. parapsilosis*), no significant increases in MIC values were observed after several weeks of drug exposure [46]. Therefore, we extended our experimental design to a total of 105 weeks. Our results validate this approach, as observable changes in MIC values for both antifungal agents emerged only after 30–45 weeks of continuous passaging. In the case of natamycin, resistant strains showing a substantial MIC increase were obtained only after 60–75 weeks.

Azoles became the second class of antifungals utilized in in vitro resistance modeling in *Candida* species, beginning in 1997, when significant reductions in fluconazole susceptibility were reported [45]. Similarly, induced resistance was achieved in *C. tropicalis* strains, where even higher MIC values were observed [47]. In contrast, Cowen et al. (2000), who used a single *C. albicans* strain clonally divided into multiple variants, observed a less pronounced reduction in fluconazole susceptibility compared to earlier studies [48]. In the study by Paul et al. (2020), resistance to fluconazole was successfully induced in *C. tropicalis*, although the final MIC values were lower than those reported in the model by Barchiesi et al. (2000) [47,49]. As with polyenes, the increase in azole resistance was accompanied by a decrease in virulence. Many recent studies have focused on inducing fluconazole resistance in *C. auris*, which is considered a serious global public health threat [50,61,62].

In the case of *Malassezia*, the number of studies investigating resistance induction is significantly lower compared to *Candida*, which motivated our focus on a less-studied yeast species that nonetheless poses a considerable challenge in veterinary medicine. In their study, Jesus et al. (2011) reported a marked increase in MIC values for fluconazole, ketoconazole, and itraconazole among 30 *M. pachydermatis* isolates [63]. Similarly, Nakano et al. (2005) observed increased MIC values for ketoconazole, nystatin, and terbinafine in this species through continuous culture in media containing concentrations close to their initial MICs [14]. In our study, we deliberately avoided using high antifungal concentrations and instead opted for sublethal doses set at 50% of the baseline MIC for each strain. This strategy aimed to minimize the risk of eliminating strains due to excessive drug pressure and to better simulate gradual adaptation. Our results validate this approach: even at reduced concentrations, several strains failed to survive until the end of the experiment. Had we used concentrations closer to the initial MICs, the number of eliminated strains would likely have been higher. Some researchers, wanting to accelerate the process of resistance development, used additional external factors. For example, in a study by Kano and Kamata (2020), elevated MIC values for ketoconazole and miconazole were obtained in *Malassezia* isolates through methods such as exposure to N-methyl-N’-nitro-N-nitrosoguanidine (MNNG), ultraviolet radiation, and subculturing on solid media supplemented with increasing antifungal concentrations [64]. These approaches intentionally introduced additional selective pressures to accelerate the emergence of resistance to azoles. Although our research concerned polyenes, when planning our experiment, we wanted to eliminate all other factors that could influence the development of resistance in order to be able to observe only the effect of the pressure of sublethal doses of selected antimycotics. By employing controlled laboratory conditions, we ensured that the observed changes were solely attributable to the selective pressure exerted by the antifungal compounds. This approach enables the real-time monitoring of resistance development and may facilitate more effective strategies to combat antifungal resistance in the future. It also allows for the identification of phenotypic and molecular alterations associated with increased tolerance to polyenes in strains that were originally susceptible.

An essential factor in the effective treatment of fungal infections may be the early detection of infections caused by pathogens with a high potential for developing antifungal resistance. Early identification of that kind of strain would allow for a precise treatment method, including the appropriate selection and dosing of antifungal agents, thereby improving treatment efficacy and reducing the risk of secondary resistance development.

The obtained model enables the tracking of structural, biochemical, and genetic changes occurring during the acquisition of resistance to polyenes in *M. pachydermatis* and may prove valuable in elucidating the mechanisms underlying the evolution of antifungal drug resistance. Furthermore, the results may help identify features present in initially susceptible strains that have potential for developing resistance. Identifying such characteristics could have significant clinical implications, particularly for screening potentially high-risk strains among common opportunistic fungi.

Polyenes represent a class of antifungal agents that exert their activity by directly binding to ergosterol in the fungal cell membrane, leading to pore formation and a subsequent loss of membrane integrity. Resistance to polyenes in yeast-like fungi, including *M. pachydermatis*, may arise through several mechanisms that are increasingly well understood owing to advances in molecular research.

One of the principal mechanisms involves a reduction in ergosterol content or structural alterations of this sterol within the cell membrane, which decreases the ability of polyenes to interact with their target site. Such modifications may result from mutations in genes of the ergosterol biosynthesis pathway, such as ERG3, ERG6, or ERG11 (Appendix A, Table A1) [65,66]. Although most studies have focused on azole resistance, mutations in these same genes can also influence polyene efficacy by modifying the cellular sterol profile [67].

A second important mechanism is the overexpression of efflux pumps, belonging to the ATP-binding cassette (ABC) and Major Facilitator Superfamily (MFS) transporter families. These membrane transporters can actively expel antifungal compounds from the cell, thereby reducing their intracellular concentration. While the contribution of efflux pumps to polyene resistance has not yet been definitively confirmed in *Malassezia* spp., evidence from other fungi (e.g., *Candida* spp.) and preliminary observations within the genus *Malassezia* suggest that they may play a significant role in multidrug resistance [68,69].

Additionally, the ability of *M. pachydermatis* to form biofilms may contribute to phenotypic resistance. Biofilms act as a physical barrier that limits drug penetration, while cells embedded within the biofilm exhibit altered metabolic activity and differential expression of resistance-associated genes [67,68].

In light of these findings, further research into the molecular basis of polyene resistance in *M. pachydermatis* is warranted. Comprehensive analyses of sterol biosynthesis gene sequences, membrane transporter expression profiles, and biofilm-related properties could provide valuable insights into the mechanisms underlying resistance and may help identify novel therapeutic targets.

## Figures and Tables

**Table 1:** Changes in MIC and MFC values of nystatin for individual *M. pachydermatis* strains over 105 weekly passages on nystatin-supplemented media (4 μg/mL); X-strain naturally eliminated during the experiment.

MIC/MFC Value of NYSTATIN [µg/mL]									
	Passage	0	15	30	45	60	75	90	105
Strain									
Z8		8/8	8/12	8/8	12/14	14/14	14/14	18/20	22/24
Z15		8/8	8/10	10/10	6/16	X/X	X/X	X/X	X/X
Z27		8/8	10/12	6/16	22/24	34/34	34/34	38/38	38/38
Z28		16/16	10/12	6/12	18/22	24/32	22/22	26/30	30/32
Z63		8/14	10/10	6/8	6/6	X/X	X/X	X/X	X/X
5PU		6/8	12/18	14/20	6/10	X/X	X/X	X/X	X/X
28PU		6/8	6/6	20/22	20/20	28/28	26/26	30/30	28/28
42PU		8/8	10/12	6/8	18/18	32/32	22/22	26/26	22/22
43PU		16/16	6/8	26/26	24/24	34/34	26/26	34/34	34/34
46PU		32/32	18/22	6/12	12/12	X/X	X/X	X/X	X/X

**Table 2:** Changes in MIC and MFC values of natamycin for individual *M. pachydermatis* strains over 105 weekly passages on natamycin-supplemented media (8 µg/mL); X-strain naturally eliminated during the experiment.

MIC/MFC Value of NATAMYCIN [µg/mL]									
	Passage	0	15	30	45	60	75	90	105
Strain									
Z8		16/22	16/26	16/16	10/12	X/X	X/X	X/X	X/X
Z15		16/16	16/16	12/18	22/24	22/22	34/34	30/30	28/28
Z27		16/28	16/28	10/10	24/28	36/36	34/34	30/30	24/24
Z28		16/16	22/30	20/20	18/18	12/12	12/12	16/16	16/16
Z63		16/28	22/30	14/24	24/30	34/36	32/32	28/28	22/22
5PU		32/32	32/34	12/26	30/34	40/44	34/40	30/34	24/24
28PU		16/28	22/32	20/20	28/32	48/48	34/34	32/32	20/20
42PU		16/16	16/20	16/28	26/30	42/48	44/44	36/38	24/24
43PU		16/16	16/18	12/20	24/26	44/48	36/36	32/32	22/26
46PU		16/28	34/34	24/24	30/32	36/36	36/36	38/38	34/34

**Table 3:** Comparison of MIC and MFC values for nystatin in *M. pachydermatis* strains at the initial state, after 105 weekly passages on nystatin-supplemented media (4 μg/mL), and after 10 additional passages on antifungal-free medium; X-strain naturally eliminated during the experiment.

NYSTATIN							
Strain	Initial State		105 Passage		Multiplicity of Initial State MIC	10 Passage Without Antymycotic	
	MIC [µg/mL]	MFC [µg/mL]	MIC [µg/mL]	MFC [µg/mL]		MIC [µg/mL]	MFC [µg/mL]
Z8	8	8	20	24	2.5	22	24
Z15	8	8	X	X	X	X	X
Z27	8	8	38	38	4.75	28	28
Z28	16	16	30	32	1.88	30	32
Z63	8	14	X	X	X	X	X
5PU	6	8	X	X	X	X	X
28PU	6	8	30	30	5	28	28
42PU	8	8	22	34	2.75	22	22
43PU	16	16	34	34	2.13	32	32
46PU	32	32	X	X	X	X	X

**Table 4:** Comparison of MIC and MFC values for natamycin in *M. pachydermatis* strains at the initial state, after 105 passages on natamycin-supplemented media (8 μg/mL), and after 10 additional passages on antifungal-free medium; X-strain naturally excluded during the course of the experiment.

NATAMYCIN							
Strain	Initial State		105 Passage		Multiplicity of Initial State MIC	10 Passage Without Antymycotic	
	MIC [µg/mL]	MFC [µg/mL]	MIC [µg/mL]	MFC [µg/mL]		MIC [µg/mL]	MFC [µg/mL]
Z8	16	22	X	X	X	X	X
Z15	16	16	28	28	1.75	20	22
Z27	16	28	24	24	1.5	24	24
Z28	16	16	16	16	1	16	16
Z63	16	28	22	22	1.38	20	20
5PU	32	32	24	24	0.75	20	20
28PU	16	28	20	20	1.25	20	20
42PU	16	16	24	24	1.5	24	24
43PU	16	16	22	26	1.38	16	18
46PU	16	16	34	34	2.13	16	16

## Data Availability

The original contributions presented in this study are included in the article. Further inquiries can be directed to the corresponding author.

## Appendix A

**Table A1:** List of candidate genes potentially involved in mechanisms of resistance to polyenes.

Gene	Protein/ Enzyme Function	Known Role in Fungal Physiology or Drug Resistance	Documented or Expected Resistance-Associated Effect	Relevance for *M. pachydermatis*	References
FAS1/FAS2	Fatty acid synthase α- and β-subunits (multifunctional enzyme complex)	Catalyze de novo synthesis of long-chain fatty acids; regulate membrane lipid composition and saturation.	Altered FA desaturation or elongation modifies membrane fluidity and polyene insertion.	Functional in *M. pachydermatis* (retained FAS cluster); potential adaptive target during AmB exposure.	[6,70]
ERG2	C-8 sterol isomerase	Converts Δ8-sterols to Δ7-sterols in ergosterol pathway.	Loss-of-function mutations yield sterol intermediates with reduced AmB affinity.	Sequencing recommended after sublethal AmB passages.	[25,71]
ERG3	C-5 sterol desaturase	Introduces C-5(6) double bond; essential for ergosterol formation.	*ERG3* mutants lack ergosterol, accumulating precursors that confer AmB resistance.	Key candidate; analogous to resistant *Candida* phenotypes.	[25,72]
ERG6	Sterol C-24 methyltransferase	Adds methyl group at C-24; regulates sterol side-chain structure.	Mutation alters sterol composition, increasing AmB tolerance.	Present in genome; potential adaptive hotspot.	[64,73]
ERG11 (CYP51)	Lanosterol 14-α-demethylase (cytochrome P450)	Catalyzes demethylation of lanosterol; major azole target.	Mutations/overexpression indirectly modify sterol pool and polyene sensitivity.	Expression profiling may reveal cross-talk between azole and polyene responses.	[65,67]
SUR4 (ELO3)	Fatty acid elongase for C26 very-long-chain FA	Generates VLCFAs used in sphingolipid/ceramide synthesis.	Disruption perturbs sphingolipid–sterol rafts, altering AmB diffusion.	Sphingolipid-rich *Malassezia* membranes make this gene highly relevant.	[74]
FEN1 (ELO2)	Fatty acid elongase (C22–C24 VLCFAs)	Works with *SUR4* in sphingolipid biosynthesis; affects membrane order.	Mutations confer AmB tolerance in *Saccharomyces*/*Candida*.	Candidate for lipidomic correlation analyses.	[25,74]
PDR5/CDR1-like	ABC-type efflux transporters	Export azoles/xenobiotics; occasionally affect polyenes.	Overexpression may lower intracellular AmB in biofilm cells.	ABC transporters annotated in *M. pachydermatis*; role minor but plausible.	[68,69]
HSP90/HSP70	Heat-shock proteins (chaperones)	Stabilize regulators of stress and cell-wall integrity pathways.	Upregulation enhances survival under drug stress; inhibition restores susceptibility.	Likely involved in transient tolerance.	[25,75]
SOD1/CAT1/GPX	Superoxide dismutase, catalase, glutathione peroxidase	Detoxify reactive oxygen species (ROS).	Enhanced antioxidant capacity mitigates AmB-induced oxidative injury.	High relevance; AmB triggers ROS production in yeasts.	[25,76]
